# Amputation Triggered by Gefitinib: An Unusual Clinical Presentation

**DOI:** 10.7759/cureus.60234

**Published:** 2024-05-13

**Authors:** Lihua Tao, Jiaqi Ruan, Xiaodong Chu, Pengfei Shan

**Affiliations:** 1 Department of Endocrinology and Rheumatology, Linping Campus, The Second Affiliated Hospital, Zhejiang University School of Medicine, Hangzhou, CHN; 2 Department of Gastroenterology, The First Affiliated Hospital, Zhejiang University School of Medicine, Hangzhou, CHN; 3 Department of Orthopedics, Linping Campus, The Second Affiliated Hospital, Zhejiang University School of Medicine, Hanzhou, CHN; 4 Department of Endocrinology and Metabolism, The Second Affiliated Hospital, Zhejiang University School of Medicine, Hangzhou, CHN

**Keywords:** side-effects, adenocarcinoma of the lung, gefitinib, lower limb amputation, diabetes mellitus type 2

## Abstract

Gefitinib is an epidermal growth factor tyrosine kinase inhibitor used as a targeted chemotherapeutic agent in the treatment of lung cancer and other solid malignancies. The most common adverse effects of gefitinib include dermatological side effects and gastrointestinal symptoms, with rare reports of vascular side effects such as myocardial infarction and stroke. We recently reported a case of a patient with diabetes and multiple comorbidities who developed a serious lower limb vascular adverse event after gefitinib treatment, ultimately leading to amputation surgery. This is the first reported case of lower extremity amputation following gefitinib therapy in a patient with type 2 diabetes mellitus and lung adenocarcinoma. This case highlights the potential risk of amputation in diabetic patients receiving targeted therapies like gefitinib, especially in those with vascular complications. It emphasizes the importance of exercising extra caution when dealing with these patients.

## Introduction

Gefitinib, an oral small-molecule epidermal growth factor receptor tyrosine kinase inhibitor (EGFR-TKI), is commonly used in the treatment of advanced non-small cell lung cancer (NSCLC). It inhibits the signaling pathway of the EGFR on tumor cells, thereby impeding the growth and spread of tumor cells. Gefitinib has shown significant efficacy in patients with NSCLC with EGFR mutations. However, several adverse effects have been reported, with the most frequent being anorexia, diarrhea, acne-like rash, and mild-to-moderate elevation of hepatic transaminases [1,2]. While cardiovascular adverse events are rarely reported, isolated cases of acute coronary syndrome and cardiomyopathy have been documented. During our literature search, we identified two cases of gefitinib-related vascular adverse events, one of which involved a patient with a history of diabetes who suffered from a myocardial infarction [1,3]. The patient in our case also had diabetes and multiple microvascular complications. Following gefitinib administration, severe vascular adverse events occurred, ultimately leading to limb amputation. We aim to investigate the potential mechanisms behind this severe outcome, enhance our understanding of the safety and side effects of gefitinib in diabetic patients, and work toward preventing similar adverse events in the future.

## Case presentation

The patient in our case was a 74-year-old woman with diabetes for over 20 years. She had poor glucose control. Her renal function tests revealed an increased serum creatinine level of 154 mmol/L and a vascular color Doppler ultrasound indicated some small atherosclerotic plaques in both of her lower limb arteries. Electromyography showed moderate peripheral neuropathy. Therefore, she was previously diagnosed with diabetic peripheral vascular disease, diabetic kidney disease, and diabetic neuropathy according to the diagnostic criteria for diabetes outlined by the American Diabetes Association (ADA) [4] in April 2020, marking her first hospitalization. After we switched her antidiabetic therapy to detemir insulin and lispro insulin, her fasting blood glucose level, as well as other diabetic complication indicators including serum creatinine level, vascular atherosclerotic plaque and feet numbness, were all under great control (fasting blood glucose around 8 mmol/L, postprandial blood sugar around 10 mmol/L, serum creatinine level around 150 mmol/L, and vascular atherosclerotic plaque did not influence arterial blood flow).

This patient kept our antidiabetic therapy and maintained regular follow-ups in our outpatient clinic. On April 26, 2020, during a routine checkup, a ground-glass nodule in the left upper lobe of her lungs was identified, which was confirmed later as lung adenocarcinoma through biopsy. She did not receive chemotherapy or surgical treatment because of her advanced age and poor pulmonary function. Genetic testing of the tumor samples revealed an EGFR exon 19 mutation, making targeted therapy an optimal choice. This patient eventually received a single targeted therapy of 0.25 g gefitinib tablets per day since May 22, 2020, for antineoplastic treatment. One month later, the patient complained about dry and peeling skin on both hands and feet, with an increasing serum creatinine level. In the following six months, her serum creatinine level kept increasing and the skin on her feet began to break and purulent. Subsequently, she was readmitted to the hospital on February 7, 2021.

**Figure 1:**
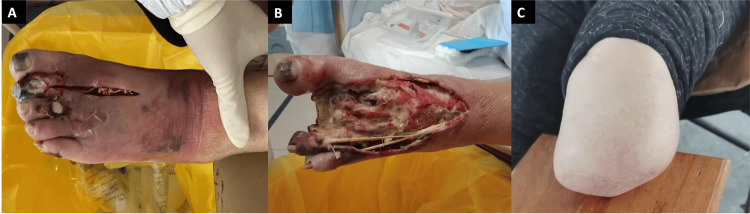
Progression of the diabetic foot after taking gefitinib. After treatment with gefitinib, the patient developed skin peeling, rupture, and an infected toe that resulted in gangrene. (A) Toe gangrene infection in February 2021, (B) the infection spreading to the entire foot in March 2021; and (C) the situation after amputation in April 2022.

The physical examination of this patient revealed dry skin on all four limbs and necrosis of the second toe on the left foot, with necrotic tissue covering the dorsal surface of the foot, exposed tendons, and purulent discharge. The skin between the toes was moist and peeling, with redness and swelling on the dorsal surface of the anterior and middle part of the left foot. Five out of 10 locations on the soles of both feet showed no response to a 10 g nylon wire. The pulsation of her dorsal artery was normal and the ankle-brachial index (ABI) was 0.9 (normal value 0.9-1.3). The laboratory examination indicated a rapidly increased serum creatinine level of 526 mmol/L, with a decreased creatinine clearance rate of 7.99 mL/minute/1.73 m^2^. The results of her laboratory tests during hospitalization are presented in Table 1. Vascular color Doppler ultrasound was performed from her femoral blood vessels to dorsal foot vessels. No venous thrombosis was detected in her lower extremities, but some small atherosclerotic plaques were found in her arteries. The largest plaque in her left lower limb artery was 0.38 cm × 0.20 cm, and in her right lower limb artery was 0.47 cm × 0.20 cm, which was similar to the sizes in April 2020 (left, 0.36 cm × 0.18 cm; right, 0.54 cm × 0.15 cm). The blood flow in her arteries was smooth, not influenced by the atherosclerotic plaques. An ophthalmic examination suggested a normal retinal vessel. According to the International Working Group on the Diabetic Foot (IWGDF) 2019 *Guidelines on the Classification of Diabetic Foot Ulcers *[5], the diabetic foot ulcer was classified as a moderate infection with a Severity of Diabetic Foot Ulceration Scoring System (SINBAD) score of 6 (Figure 1A). The patient was additionally diagnosed with a diabetic foot infection (SINBAD 6) this time.

**Table 1:** Participant characteristics during hospitalization (February 2021). WBC, white blood cell; HGB: hemoglobin; CRP, C-reactive protein; ALT, alanine transferase; ALB, albumin; BUN, urea nitrogen; Cr, creatinine; Ccr, creatinine clearance; FBS, fasting glucose; PBG, postprandial blood glucose; HbA1c, hemoglobin A1c; TG, triglyceride; LDL, low-density lipoprotein cholesterol; CYFRA21-1, cytokeratin-19-fragment; SCC, squamous cell carcinoma antigen; BP, blood pressure

	Test results	Normal values
WBC	21.8 × 109/L	3.5-10 × 10^9^/L
HGB	60 g/L	113-151 g/L
Platelet	191 × 10^9^/L	150-350 × 10^9^/L
CRP	281 mg/dL	<10 mg/dL
ALT	14 U/L	<42 U/L
ALB	23.4 g/L	37-53 g/L
BUN	97.89 mg/dL	8-20 mg/dL
Cr	526 mmol/L	45-90 mmol/L
Ccr	7.99 mL/minute	80-120 mL/minute
FBS	3.63 mmol/L	3.9-6.1 mmol/L
PBG	7.9 mmol/L	<7.8 mmol/L
TG	1.07 mmol/L	<1.69 mmol/L
LDL	1.51 mmol/L	<3.4 mmol/L
HbA1c	5.10%	4.7%-8.5%
CA125	73.5 U/mL	<35 U/mL
SCC	9.5 ng/mL	<1.5 ng/mL
CYFRA21-1	2.55 ng/mL	<4.1 ng/mL
BP	115/51 mmHg	90-139/60-89 mmHg

Since the patient demonstrated ideal control over blood glucose and had a relatively good lower limb blood flow since April 2020, diabetic peripheral vasculopathy was not considered the trigger of her foot ulcer. We also ruled out other possibly relevant factors that could lead to her foot ulcers like mechanical injuries, cold injuries, and burns. Eventually, the gefitinib-induced microcirculatory dysregulation was considered the main contributing factor to her foot ulcer and suddenly increasing Cr level. Gefitinib was discontinued on February 8, 2021. For the infection in the left lower limb, a secretion sample was collected for culture, and local wound debridement was performed. The patient received intravenous levofloxacin 0.4 g once daily, which was later adjusted to imipenem-cilastatin 500 mg every eight hours based on the results of antimicrobial susceptibility testing. However, the infection could not be controlled, leading to systemic inflammation and cardiac and renal dysfunction. Blood dialysis, blood transfusion, and albumin supplementation were performed. Despite improvements in cardiac and renal function, the infection had spread to the deep fascia and bone marrow, making healing difficult (Figure 1B). A decision was made by the multi-disciplinary team, and the patient underwent a major below-the-knee amputation of her left lower extremity on April 22, 2021 (her left leg was amputated below the knee).

Over two years of follow-up post-amputation, the patient did not receive further treatment with gefitinib. There was no skin peeling, and the toes and limb amputated stumps did not develop further necrosis or infection (Figure 1C). Fasting blood glucose levels varied from 6 to 8 mmol/L, and glycated hemoglobin levels were approximately 7%. Renal function improved, with a serum creatinine level consistently around 160 mmol/L. Cardiac function also improved, and the patient was able to carry out daily activities independently. However, the size of the pulmonary mass increased from 16 mm × 12 mm when gefitinib was stopped to 23 mm × 31 mm.

## Discussion

We present a case of a diabetic patient who underwent amputation after being treated with the EGFR-TKI gefitinib. The vascular adverse events of EGFR-TKI, especially gefitinib, are relatively rarely reported. A meta-analysis involving 17,800 patients showed that the most common adverse reactions in patients receiving EGFR-TKIs were skin and gastrointestinal reactions, with only 6% of patients experiencing cardiovascular events [6]. A retrospective study specifically analyzed the correlation between cardiovascular events and EGFR-TKIs [7]. The first-generation EGFR-TKI, erlotinib, had a higher incidence of thromboembolic and coronary artery events compared to gefitinib, which only had two cases of vascular adverse events [1,3]. This is the first reported case of amputation related to gefitinib treatment.

In this case, we suspect the diabetic foot and following amputation were related to gefitinib treatment for two reasons. First, as an EGFR-TKI, gefitinib carries a potential risk of causing skin damage and exacerbating microcirculatory disorders, which are two important risk factors of foot gangrene. Studies have shown that EGFR is involved in the growth and repair of endothelial cells, and the inhibition of EGFR can block the migration of endothelial cells, interfering with the repair and healing of damaged endothelial cells, especially in vessels with pre-existing endothelial cell injury [8]. Additionally, gefitinib affects the coagulation process by enhancing the generation of thromboxane A2 in adenosine diphosphate (ADP)-induced activated platelets, promoting thrombus formation [3]. These effects exacerbate preexisting microcirculatory disorders, making tissue cells more prone to ischemic necrosis. EGFR inhibition can also disrupt the skin barrier by causing abnormal proliferation, migration, and differentiation of skin keratinocytes [9], as well as triggering inflammatory reactions in the skin. These reactions can lead to the release of inflammatory and chemotactic factors, impacting the production of antimicrobial peptides and skin barrier proteins, thereby compromising the skin's defense against microorganisms [10]. In our case, after taking gefitinib for one month, our patient experienced dry and peeling skin, which was the most common skin adverse event of EGFR inhibitor [2]. She also experienced symptoms of microcirculatory disturbance, manifested by decreased skin temperature, darkened skin color of distal lower limbs, and gradually increased serum creatinine levels. These symptoms mentioned above could all be related to gefitinib theoretically.

The second reason for us to think of gefitinib but not diabetes itself was the trigger of her foot ulcer was her diabetic vasculopathy at that time was not severe enough to cause such serious microcirculation disturbance during that period. Our patient demonstrated ideal control over blood glucose, blood pressure, and lipid levels ever since we adjusted her antidiabetic therapy in April 2020. Although she did show diabetic vascular complications before gefitinib treatment, her dorsal artery pulses were palpable during her hospitalization in February 2021, and the sizes of her atherosclerotic plaques did not increase, meaning her macrovascular complications were not serious. Also, her retinal vessels were normal according to ophthalmic examination, which was inconsistent with her rapidly increased creatinine level if it was caused by diabetic microangiopathy. Therefore, we thought diabetes or diabetic peripheral vascular disease alone could not explain the rapid creatinine elevation and sudden foot gangrene after the use of gefitinib. Another supporting evidence was that after stopping gefitinib, the patient showed no more skin peeling or gangrene on distal limbs. Her serum Cr level was also back to 160 mmol/L.

The amputation of our patient after the use of gefitinib shows the possible serious vascular adverse events of gefitinib. It is worth noting that the change in microcirculation is particularly pronounced in patients with existing vascular disease. Studies [11] on a similar medication, nilotinib, have also indicated that patients with pre-existing vascular disease are more likely to experience microcirculation disorders and amputations after treatment with nilotinib. Therefore, physicians should comprehensively evaluate the patient's vascular complications before making treatment decisions, to ensure more rational and safe choices.

Another issue that demands our attention is the potential emergence of drug-induced skin injuries as a new contributing factor to diabetes-related amputations, given the increasing prevalence of diabetes and tumors. The primary cause of diabetic foot amputations is typically skin injuries resulting from physical factors like cuts, burns, and pressure injuries. However, our case indicates that drug-induced skin injuries can also lead to severe foot gangrene in diabetic patients. It is crucial to recognize that skin adverse reactions caused by gefitinib could go unnoticed in patients with diabetes and neuropathy due to sensory deficits that may prevent prompt detection of skin injuries or infections. Therefore, for diabetic patients receiving treatment with gefitinib or similar drugs, close monitoring of skin adverse reactions is imperative.

## Conclusions

In conclusion, gefitinib is rarely associated with clinically relevant vascular events. However, patients with diabetes mellitus and multiple vascular issues may be at a heightened risk of developing diabetic foot ulcers due to skin side effects from gefitinib-like drugs. These medications can worsen microcirculatory disorders by affecting vascular endothelial cells and promoting thrombosis, potentially leading to severe vascular adverse events such as amputation. Therefore, endocrinologists and oncologists must evaluate the vascular complications of diabetes mellitus before prescribing gefitinib-like drugs. For patients with significant vascular complications, it is essential to safeguard skin integrity and closely monitor indicators of microcirculation changes, such as serum creatinine, urine protein levels, distal limb color, and temperature. Early assessment and prompt treatment can help improve disease management and prevent the progression of complications, ultimately reducing the risk of severe vascular adverse events.
